# Understanding the Consequences of Repetitive Subconcussive Head Impacts in Sport: Brain Changes and Dampened Motor Control Are Seen After Boxing Practice

**DOI:** 10.3389/fnhum.2019.00294

**Published:** 2019-09-10

**Authors:** Thomas G. Di Virgilio, Magdalena Ietswaart, Lindsay Wilson, David I. Donaldson, Angus M. Hunter

**Affiliations:** ^1^Physiology, Exercise and Nutrition Research Group, University of Stirling, Stirling, United Kingdom; ^2^Psychology, Faculty of Natural Sciences, University of Stirling, Stirling, United Kingdom

**Keywords:** subconcussive head impacts, TBI, transcranial magnetic stimulation, motor unit behavior, boxing

## Abstract

**Objectives:**

The potential effects of exposure to repetitive subconcussive head impacts through routine participation in sport are not understood. To investigate the effects of repetitive subconcussive head impacts we studied boxers following customary training (sparring) using transcranial magnetic stimulation (TMS), decomposition electromyographic (EMG) and tests of memory.

**Methods:**

Twenty amateur boxers performed three 3-min sparring bouts. Parameters of brain function and motor control were assessed prior to sparring and again immediately, 1 h and 24 h post-sparring. Twenty control participants were assessed following mock-sparring.

**Results:**

One hour after sparring boxers showed increased corticomotor inhibition, altered motor unit recruitment strategies, and decreased memory performance relative to controls, with values returning to baseline by the 24 h follow up.

**Conclusion:**

Repetitive subconcussive head impacts associated with sparring resulted in acute and transient brain changes similar to those previously reported in soccer heading, providing convergent evidence that sport-related head impacts produce a GABAergic response. These acute changes in brain health are reminiscent of effects seen following brain injury, and suggest a potential mechanism underlying the damaging long-term effects of routine repetitive head impacts in sport.

## Introduction

Across the sporting world there is growing concern about long-term health outcomes of traumatic brain injury. Recent evidence suggests that repeated exposure to both concussive (Wilson et al., 2017) and subconcussive head impacts (Smith et al., 2013; McKee et al., 2014, Ling et al., 2017) can affect long-term brain health. To date, relatively little attention has been paid to understanding mechanisms by which these chronic effects arise. In principle, however, the origins of long-term damage must first be evident in acute brain changes. The focus of the current paper is therefore to characterize the acute brain alterations that occur following routine head impacts in sport.

Direct evidence for brain and brain-to-muscle changes following subconcussive impacts comes from our previous work suggesting increased GABAergic activity following a single soccer heading drill (Di Virgilio et al., 2016). More recently, soccer heading has been found to elevate fluid markers of neural damage (Wallace et al., 2018; Wirsching et al., 2019), further supporting the idea that sport-related head impacts produce acute brain impairments. However, whilst soccer heading has proved to be an excellent model for revealing the acute sub-clinical consequences of routine head impacts in sport, the link between soccer heading and increased risk of degenerative disease is not yet widely accepted. Consequently, here we turn to an alternative sport – boxing – wherein the direct link between head impacts, and poor long-term brain health is well documented (Jordan et al., 1992a, b, 1996; Stiller et al., 2014). Boxing also allows us to study naturalistic exposure to repeated head impacts: we assessed brain function before and after boxers performed their standard sparring training.

The nature of the physiological and cognitive changes found following soccer heading (Di Virgilio et al., 2016) suggests that sport-related head impacts produce a GABAergic response, the neurotransmitter involved in the inhibitory pathways (Inghilleri et al., 1993). Therefore, our approach is to use transcranial magnetic stimulation (TMS) to evaluate corticomotor inhibition, which is known to be GABA dependent (Kobayashi and Pascual-Leone, 2003). Perhaps the most direct evidence of TMS assessed corticomotor inhibition being a measure of changes in GABA comes from pharmacological interventions, showing that administering a GABA_B_ receptor agonist (baclofen) increases cSP without affecting spinal inhibitory mechanism (Siebner et al., 1998; Stetkarova and Kofler, 2013). Changes in the GABAergic response are evident in a lengthening of the cortical silent period and are associated with acute changes in other GABA dependent behaviors, including memory impairments and motor control. Critically, each of these dimensions has been shown to be affected by brain damage produced by concussion (Major et al., 2015) and stroke (Classen et al., 1997), providing converging evidence that GABAergic response may be a mechanism that bridges acute and chronic effects of impacts to the head.

The current study also allows us to examine an additional potential GABAergic response related to changes in motor control (Nishimaru and Kakizaki, 2009; Gowda et al., 2018), assessed through the use of precision decomposition electromyographic (dEMG). Increased GABAergic inhibitory activity should dampen the neural drive to the muscles, resulting in a measurable reduction in motor unit firing rate (Watanabe et al., 2002). Changes in motor control have been demonstrated following subconcussive head impacts (Haran et al., 2013; Kawata et al., 2016; Hwang et al., 2017) and concussion, specifically in balance (Powers et al., 2014), gait (Doherty et al., 2017) and vestibular function (Cheever et al., 2018). Evidence for acute impairments in neuromuscular control is particularly important because it may also explain why athletes returning to play following a concussion are more susceptible to musculoskeletal injuries than a healthy population (Lynall et al., 2015; Cross et al., 2017).

To characterize the acute brain changes that occur following routine head impacts in sport, this study assesses brain function before and after a single boxing sparring session. We predict that sparring will increase corticomotor inhibition and decrease long-term memory function (i.e., learning). In addition, we expect the underlying heightened GABAergic response to produce altered motor unit recruitment patterns, providing evidence that sport-related head impacts also produce acute changes in motor behavior.

## Methodology

### Approvals and Recruitment

Twenty healthy, amateur boxers and Muay Thai athletes from University boxing and Muay Thai clubs (age 22 ± 1.7 years; mass 76 ± 7.5 kg; height 178 ± 8.4 cm; sparring experience 3 ± 1.6 years) and 20 age-matched controls (age 22 ± 3 years; mass 76.4 ± 13.6 kg; height 174.8 ± 9.8 cm) were recruited for the study. Participants were excluded from taking part if they presented with any of the following: (1) history of traumatic brain injury resulting in loss of consciousness; (2) history of a neurological condition; (3) clinically diagnosed concussion in the 12-months prior to taking part. The local research ethics committee approved the study, and procedures conformed to the guidelines set out by the Declaration of Helsinki. Written informed consent was obtained from all participants.

### Study Design

Prior to the first experimental trial participants attended a familiarization session to minimize the possibility of learning effects. Participants were asked to refrain from vigorous physical activity, consuming alcohol and caffeine or smoking for 24 h prior to each trial. Participants reported to the laboratory following an overnight fast, and provided with a standardized breakfast (30 g cornflakes, 150 ml semi-skimmed milk). Parameters of cognitive function, postural control, corticospinal excitability, corticomotor inhibition and MU firing instances were recorded at baseline and immediately, 1 h and 24 h following a sparring session. The control group only completed the first phase of the study (corticomotor control and cognitive function) and did not undergo testing at the 24 h follow-up.

### Sparring Protocol

The sparring session consisted of 3 × 3-min rounds with 2 min rest in between each round. Participants provided their own sparring partner and boxing equipment, and were instructed to spar as in a normal training session. The control group simulated a sparring session; each mock-sparring round started with a 10 s period of moving about, followed by a 20 s bout of pad hitting using a 3-punch combo (Right, Left, Right; Left, Right, Left; Right, Left, Right; etc.); cues for each combination were given by a metronome set at 30 bpm. Participants alternated between moving about and pad hitting until the 3-min round was up, completing a total of 180 pad-hits. The mock-sparring session was specifically intended to emulate levels of activity observed in real sparring. Feedback from athletes during pilot testing was used to refine the metronome paced tempo and pattern of punching to ensure the control condition was well matched to the demands of sparring practice.

### Transcranial Magnetic Stimulation

Motor evoked potentials (MEPs) were elicited in the rectus femoris (RF) of the dominant leg via single pulse TMS and assessed using electromyographic (EMG) recordings. Single magnetic stimuli of 1 ms duration were applied over the contralateral primary motor cortex (M1) using a magnetic stimulator (Magstim 2002 unit, The Magstim Company Ltd., Whitland, United Kingdom) and a 110 mm double-cone coil. Optimal coil location for generating MEPs was determined by placing the coil over M1, laterally to the vertex; the area where the largest MEP peak-to-peak amplitudes occurred was identified and marked on the scalp. The active motor threshold (aMT) for the RF was determined by increasing stimulator output from 10% by 5% increments, while the participant held a ∼20% maximal voluntary isometric contraction (MVC) until discernible MEPs were visible (Wilson et al., 1995). Once aMT was established, subsequent stimulations were delivered at 130% aMT.

MEPs, alongside all other EMG measures, were recorded with participants sitting with their dominant leg secured to a calibrated load cell of an isokinetic dynamometer (Biodex System 4, New York, NY, United States), with knee angle set at 60° (0 = fully extended limb). To assess corticomotor inhibition participants were required to perform 3 MVCs of 5 s duration (60 s rest in-between) while a single TMS stimulation was delivered over M1. Corticomotor inhibition was quantified as the cortical silent period (cSP) duration, from the stimulation artifact to the resumption of discernible, uninterrupted EMG activity.

For corticospinal excitability, participants maintained a 20% MVC while 20 single TMS pulses, separated by 6 s, were delivered over M1. Corticospinal excitability was determined as the average MEP amplitude normalized to the maximal response elicited by motor nerve stimulation (%Mmax).

### Electromyography and Femoral Nerve Stimulation

Electromyographic was performed using a wireless system (Biopac Systems, Inc., Goleta, CA, United States). Data were sampled at 2 kHz, and filtered using 500 Hz low- and 1.0 Hz high-band filters. Signals were analyzed offline (Acqknowledge, v3.9.1.6, Biopac Systems, Inc., Goleta, CA, United States). EMG activity was captured using Ag/AgCl surface electrodes (Vermed, Devon, United Kingdom) with an intra-electrode distance of 2 cm positioned over RF; prior to electrode placement, the area was shaved and abraded as per standard practice. The position of each electrode was marked with ink to ensure consistent placement during subsequent visits.

Stimulation of the femoral motor nerve was performed using an electrical stimulator (DS7A, Digitimer Ltd., Hertforshire, United Kingdom). The stimulation site was identified by locating the femoral artery and placing a self-adhesive surface electrode (cathode) lateral to it, high over the femoral triangle, with the anode on the gluteus maximus. Single stimuli were delivered to the muscle while participants maintained a 20% MVC, and the intensity of stimulation was increased until a plateau in twitch amplitude and RF M-wave (Mmax) occurred. Supramaximal stimulation was delivered by increasing the final stimulator output intensity by a further 30%.

### Precision Decomposition EMG

Rectus femoris surface EMG for decomposition analysis (dEMG) was measured during a 60% MVC, using a modified Bagnoli 16-channel EMG system (Delsys, Boston, MA, United States) (Hu et al., 2013). The software (EMGworks Acquisition V4.3.0) recorded four EMG signals at a sampling rate of 20 kHz, filtered with a bandwidth of 20–1750 Hz. Signals collected during this task were analyzed using dedicated software (dEMG Analysis, v 1.1.3) and Precision Decomposition III (PD III) algorithms to accurately and reliably (≥90%) decompose the raw EMG signals into individual MU action potential (MUAP) trains (Hu et al., 2013). Two out of the 19 participants for the sparring group were not included in the dEMG analysis due to poor EMG data. Recruitment thresholds for each MU were calculated as force (%MVC) relative to the first firing instance (Ye et al., 2015).

### Cognitive Function

Cognitive function was assessed using the Cambridge Neuropsychological Test Automated Battery (CANTAB, Cambridge Cognition, Cambridge, United Kingdom). The following CANTAB outcomes were included: total error score adjusted on paired associate learning (PAL) a measure of long-term memory and spatial working memory (SWM) between errors, an assessment of short-term memory.

### Postural Control

Postural control was assessed using a force platform (Bertec forceplate model 6090-15, Bertec Corporation, Columbus, OH, United States) connected to in-house software. The interface was composed of a cross hairs display with the center of the display being the center of the force platform. The device returned a value indicating the participants’ centre of pressure (COP). Participants completed 4, 20 s conditions twice: 2 legs – eyes open, 2 legs – eyes closed, 1 leg – eyes open and 1 leg – eyes closed.

### Statistical Analysis

Statistical analysis and graph creation was carried out using GraphPad 6 Prism (v 6.0; GraphPad Software, Inc.). Two-way repeated measures ANOVAs (time 3 × group 2) were used on corticomotor control and cognitive function. Recovery on each of the measures following sparring (including 24 h follow up) was analyzed through one-way repeated measures ANOVA. If a significant difference was observed, Tukey’s *post hoc* test was used to explore the effect further. When required, percentage changes were calculated by computing the difference between two measurements, dividing the difference by the baseline measurement and multiplying the answer by 100. Statistical significance was set at a *p*-value ≤ 0.05; data are presented as means (± standard deviation) unless otherwise stated.

## Results

### Corticomotor Inhibition and Corticospinal Excitability

**FIGURE 1:**
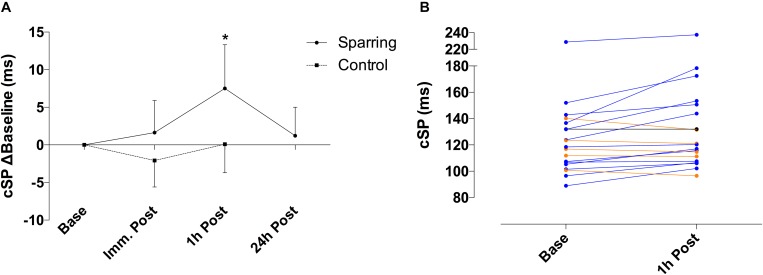
**(A)** Difference of cSP for sparring and control groups, relative to baseline. Inhibition appeared increased following sparring, peaking at the 1 h mark with a 6% increase, and returning to pre-values by the 24 h follow up. ^∗^*p* < 0.002 following sparring; error bars indicate 95% CI. **(B)** Change in cSP duration for each participant from baseline to 1 h following sparring.

**FIGURE 2:**
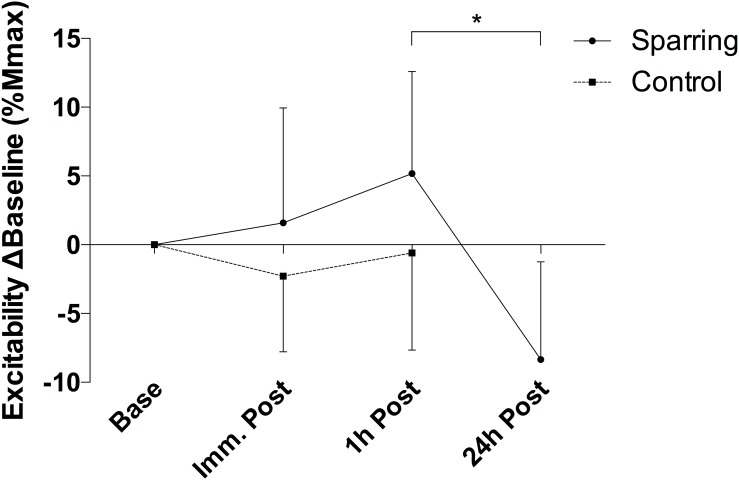
Difference of corticospinal excitability for sparring and control groups, relative to baseline. Excitability appears slightly increased immediately and 1 h post sparring (though not significant). ^∗^*p* < 0.05; error bars denote 95% CI.

Corticomotor inhibition cSP showed a significant effect of time (*p* = 0.01; *F*_(__2__, 72__)_ = 4.21) and an interaction (*p* = 0.04; *F*_(__2__, 72__)_ = 3.14) effect with the sparring and control groups (Figure 1). Corticomotor inhibition increased by 6% at the 1 h follow up in the sparring group, compared to a 0.08% increase in the control group (*p* = 0.002; 95% CI = 2.50 to 14.12). No significant effects were observed for corticospinal excitability following sparring and control exercise relative to baseline (Figure 2). Including the 24 h follow up in the analysis for the sparring group showed that whilst cSP was significantly increased 1 h following sparring (*p* = 0.03; 95 CI = 0.003 to 0.111), the values returned to baseline the following day. Excitability was significantly suppressed 24 h following sparring when compared to the 1 h follow-up (*p* = 0.014; 95% CI = 2.40 to 24.64).

### Precision Decomposition EMG

**FIGURE 3:**
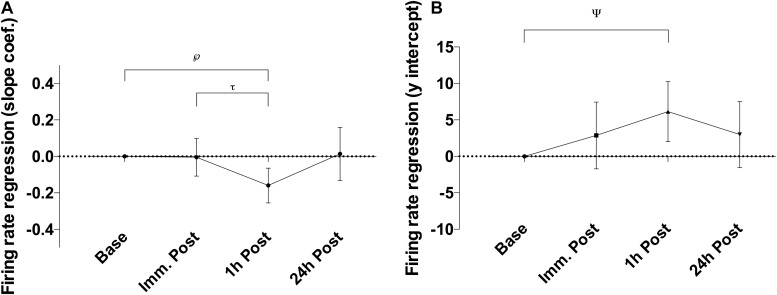
Difference of the slope coefficient **(A)** and y-intercept **(B)** of the firing regression relative to baseline. There appeared to be negative shift in the regression following sparring, peaking at the 1 h time-point. ℘*p* = 0.011; τ*p* = 0.008; ψ*p* = 0.026; error bars indicate 95% CI relative to baseline.

There was a significant alteration in the relationship between average motor unit firing rate and motor unit recruitment threshold between slope coefficient and y-intercept (*p* = 0.028; *F*_(__2__.__21__, 39__.__79__)_ = 3.76 and *p* = 0.035; *F*_(__2__.__88__, 51__.__85__)_ = 3.13, respectively – Figure 3) as a function of time, caused by a decline in the slope coefficient 1 h post sparring compared to baseline (*p* = 0.011; CI = −0.29 to −0.03) and immediately post sparring (*p* = 0.008; CI = −0.27 to −0.04 – Table 1). The y-intercept increased 1 h post sparring compared to baseline (*p* = 0.026; CI = 0.61 to 11.68 – Table 1). Both parameters returned to baseline by 24 h.

**TABLE 1:** Pairwise comparisons (mean difference, 95% confidence intervals and adjusted *p*-values) of the slope coefficient and y-intercept (rec. thresh. Vs. MUFR).

	**Tukey’s multiple**	**Mean**	**95% CI**	**Adjusted**
	**comparisons test**	**diff**	**of diff.**	***p*-value**
Slope coefficient	Imm. Post vs. Pre	–0.005	−0.14 to 0.13	0.9996
	**1 h Post vs. Pre**	−**0.160**	−**0**.**29 to**−**0**.**03**	**0.0119**
	24 h Post vs. Pre	0.014	−0.1817 to 0.21	0.9972
	**1 h Post vs. Imm. Post**	−**0.155**	−**0**.**27 to**−**0**.**04**	**0.0087**
	24 h Post vs. Imm. Post	0.018	−0.18 to 0.22	0.9936
	24 h Post vs. 1 h Post	0.173	−0.03 to 0.38	0.1130
Y-intercept	Imm. Post vs. Pre	2.868	−3.30 to 9.04	0.5662
	**1 h Post vs. Pre**	**6.147**	**0**.**61 to 11**.**68**	**0.0265**
	24 h Post vs. Pre	2.989	−3.11 to 9.09	0.5236
	1 h Post vs. Imm. Post	3.278	−1.72 to 8.28	0.2826
	24 h Post vs. Imm. Post	0.121	−5.52 to 5.76	>0.9999
	24 h Post vs. 1 h Post	–3.157	−8.67 to 2.36	0.3938

*Significant differences are highlighted in bold. Imm, Immediately.*

### Cognitive Function

**FIGURE 4:**
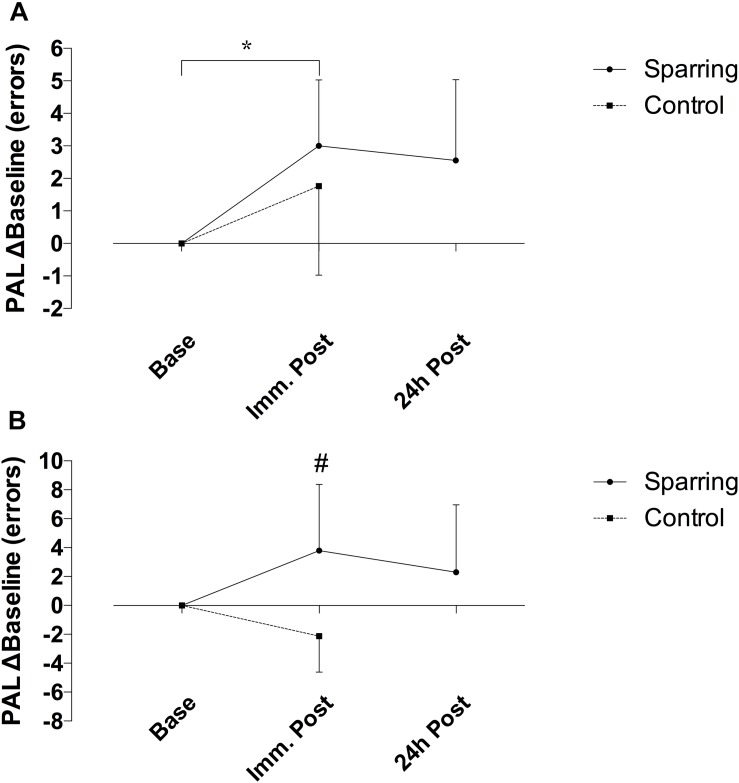
Difference in cognitive performance following sparring or control exercise, relative to baseline. **(A)** PAL performance was significantly lower following sparring compared to baseline (*p* = 0.01), although there was no interaction effect. **(B)** SWM showed an interaction effects between groups (*p* = 0.002), though not significantly different from baseline.

Performance on the CANTAB SWM task showed no effect of time but a significant interaction between time and the sparring and control groups (*p* = 0.03; *F*_(__1__, 34__)_ = 5.05) (Figure 4). The number of errors increased by 52% following sparring compared to a 28% decrease following the control exercise (*p* = 0.02; 95% CI = −10.70 to −0.68). Errors on PAL task increased over time (*p* = 0.005; *F*_(__1__, 34__)_ = 8.87). Although this effect did not differentiate the groups, further analysis of change in the sparring group between baseline, immediate outcome, and 24 h follow-up (Figure 4) showed that errors made on the PAL task increased by 47% following sparring (*p* = 0.01; 95% CI = 0.10 to 1.22). The parameter also demonstrated a tendency to remain elevated at the 24 h follow-up, a pattern not found for performance on the SWM task.

### Postural Control

Postural control was unaltered for all conditions (*p* > 0.05) (Table 2).

**TABLE 2:** Mean (± *SD*) values for each of the outcome measures: corticomotor inhibition (cortical silent period in ms) and corticospinal excitability (MEP amplitude normalized to femoral nerve M-wave, %Mmax), paired associate learning (PAL errors), spatial working memory (SWM errors), dEMG (rec. thresh. Vs. MUFR) and postural control (center of pressure for each condition) measured at each time point.

		**Assessment Time Post Sparring/exercise**		
		
**Variable**	**Baseline**	**Immediately**	**1 h**	**24 h**
**TMS**				
Sparring Inhibition (ms)	124.5 ± 30.6	126.2 ± 34.1	132.0 ± 32.9^∗^	125.7 ± 29.4
Control Inhibition (ms)	122.6 ± 13.1	120.6 ± 12.8	122.7 ± 13.9	–
Sparring Excitability (%Mmax)	50.5 ± 20.6	52.1 ± 18.8	55.7 ± 19.0	42.2 ± 19.3^#^
Control Excitability (%Mmax)	79.2 ± 38.9	76.9 ± 41.0	78.7 ± 36.3	–
**Cognitive function**				
Sparring PAL (errors)	3 ± 3	6 ± 5∂	–	5 ± 4
Control PAL (errors)	3 ± 3	5 ± 5	–	–
Sparring SWM (errors)	6 ± 9	10 ± 10β	–	9 ± 9
Control SWM (errors)	8 ± 7	5 ± 5Ω	–	–
**Rec. thresh. Vs. MUFR**				
Slope coefficient	−0.35 ± 0.20	−0.35 ± 0.16	$-0.51\pm 0.22_{℘}^{\tau }$	−0.33 ± 0.32
Y-intercept	23.99 ± 8.60	26.86 ± 7.92	30.13 ± 9.79ψ	26.98 ± 10.95
R^2^	0.72 ± 0.20	0.75 ± 0.12	0.77 ± 0.09	0.78 ± 0.14
**Postural control**				
2LEO (COP)	48.3 ± 18.6	44.8 ± 23.4	–	50.1 ± 18.2
2LEC (COP)	46.6 ± 16.5	48.8 ± 22.9	–	50.0 ± 18.1
1LEO (COP)	29.2 ± 14.8	34.6 ± 12.8	–	35.1 ± 14.3
1LEC (COP)	34.1 ± 15.4	38.3 ± 19.7	–	34.5 ± 15.2
**Force production**				
Sparring MVC	227.2 ± 63.7	220.5 ± 56.3	212.1 ± 57.2¥	226.0 ± 65.7
Control MVC	213.0 ± 70.4	206.5 ± 65.6	200.7 ± 63.1¥	–

*^∗^*p* = 0.03 Baseline v l h Post sparring; ^#^*p* = 0.01 l h Post v 24 h Post sparring; *dp* = 0.01 Baseline v Imm. Post sparring; (β*p* = 0.03 sparring v control; Ω*p* = 0.02 Baseline v Imm. Post control; ℘*p* = 0.01 Baseline v l h Post sparring; τ*p* = 0.008 Imm. Post v l h Post sparring; ψ*p* = 0.026 Baseline v l h Post sparring.; ¥*p* < 0.001 Baseline v l h Post.*

### Maximal Voluntary Contraction

Maximal voluntary contraction (MVC) significantly (*p* < 0.001; *F*_(__2__,__36__)_ = 14.3) declined by ∼6% across the 3 time points (pre, immediately and 1 h post) for both sparring and control groups but there were no difference between the groups (Table 2).

## Discussion

The aim of this study was to better understand potential effects of exposure to repetitive subconcussive head impacts through routine participation in sport. We hypothesized that repeated sport-related routine head impact produces multi-modal brain changes suggestive of a GABAergic response. This prediction was based on our earlier work (Di Virgilio et al., 2016), and emerging evidence that an imbalance between inhibition and excitation underlies the chronic consequences of TBI (Guerriero et al., 2015). To investigate this hypothesis, we examined the effects of repetitive subconcussive head impacts in boxers following customary training (sparring) using TMS, decomposition EMG and tests of memory.

The key finding of the current study is evidence for transient electrophysiological and cognitive changes following a single sparring session in a cohort of amateur boxers, mirroring the effect we previously found following a single session of football heading (Di Virgilio et al., 2016). More specifically, repeated subconcussive head impacts were associated with increased corticomotor inhibition. Behaviorally, learning and memory parameters also decreased following sparring. As an innovation, further to our previous findings, we also demonstrate dampening of motor control: the force thresholds at which early recruited motor units became active were delayed, accompanied by a decreased activation threshold for late recruited motor units.

The combined outcome measures of the current study provide converging evidence that subconcussive impacts are associated with a GABAergic response. Our findings of a heightened inhibitory response, altered motor unit recruitment, and reduced memory and learning in the sparring group and not in the controls, suggest that routine sport-related head impacts disrupt GABA signaling pathways. Dysfunction in GABA signaling is core to many common neurological and psychiatric conditions (Ramamoorthi and Lin, 2011; Clarke et al., 2012) and circuit dysfunction is thought to be key in understanding the link between brain trauma and future brain health (Dulla et al., 2016). A delicate balance between excitatory and inhibitory neurotransmission normally exists, and it is therefore of significance if homeostasis is repeatedly disrupted as a result of routine impacts in sport. Altered GABAergic transmission as a result of impacts has an influence on brain function, inhibition, and plasticity of cortical networks, as confirmed by the multi-methods evidence of this study.

A strength of the current study is that it addresses causal effects through a highly controlled experimental paradigm and by directly examining change. At the same time, by design the study evaluates the GABAergic response indirectly, examining the downstream consequences of changes in GABA. Recent developments would allow more direct examination of *in vivo* changes in GABA activity using proton spectroscopy magnetic resonance brain imaging. However, the quantification of small changes to GABA activity are methodologically challenging. Animal studies confirmed the acute metabolic response to impact, showing that 1 h after actual traumatic brain injury the balance between excitatory and inhibitory neurotransmitters is altered (Harris et al., 2012). The chronic GABAergic response in sport was confirmed using spectroscopy in boxers in this issue (Kim et al., 2019), and previously in American football players by Tremblay et al. (2014) assessed through spectroscopy as well as TMS assessed cortical silent period. They found a complex but interesting pattern of results suggesting that indeed the normal balance between GABA mediated inhibition and excitation was altered in the players who sustained a concussion on average 3 years prior. Interestingly, that same study found that subconcussion has a similar effect. Tremblay et al. (2014) found that active American Football players who did not sustain concussion also showed a lack of the excitatory and inhibitory metabolic relationships that would normally be expected based on the existing spectroscopy literature. Their findings, therefore, add weight to our hypothesis of GABAergic dysfunction following repeated subconcussive impact, in the same way as it is expected following concussive impact.

Further to quantifying the actual levels of GABA in response to impact through magnetic resonance spectroscopy, the next most direct evidence for a GABAergic response comes from increases in the cortical silent period, as used in the current study. This GABA mediated mechanism was previously found to be prolonged following (repeated) concussion (Chistyakov et al., 2001; De Beaumont et al., 2007, 2009, 2011; Tremblay et al., 2011; Miller et al., 2014; Pearce et al., 2014, 2015). Until the publication of our study in 2016 (Di Virgilio et al., 2016), the sub-clinical effects of subconcussion, particularly in the acute phase, had not yet been quantified (Pearce, 2016). The prolonged cortical silent period reported here 1 h after sparring in boxers confirms that repeated subconcussive head impact gives rise to cortical inhibitory-excitatory imbalance.

GABA mediated inhibition is critical for cognition, and memory and learning in particular, which were found to be affected by routine impacts in the current study. Increases in GABA-related inhibition are known to impair the plasticity necessary for learning, for example as demonstrated using TMS (Ziemann et al., 2001). For both memory and learning, plasticity within the neurocortex is critically dependent of GABA modulation (Stagg et al., 2011). Memory processes involving learning have been found to be impaired with increased GABA activity (Chapouthier and Venault, 2002; Yoshiike et al., 2008). This is in line with the finding reported here, demonstrating that paired associated learning error rates increased in the sparring group compared to baseline and 24 h follow up. The same prediction is perhaps less straightforward in explaining the observed decreases in working memory, as its relation to a GABAergic response is complex (Yoon et al., 2016). Performance on the short-term working memory task involves strategy use, and impairment is associated with damage to the dorsolateral prefrontal cortex (Manes et al., 2002). We previously observed transient decreases in performance on both of the cognitive tasks used in this study following football heading (Di Virgilio et al., 2016). Similarly, learning and visual memory were previously found to be impaired in elite Australian Rules footballers when compared to amateur players (Pearce et al., 2014), and overall declines in cognitive function are reported in soccer and other sports (Echemendia et al., 2001; Lipton et al., 2013). The findings of the current study therefore suggest common mechanisms may underly brain changes following concussive and repeated subconcussive impact, which we explain as related to a GABAergic response.

Whilst the present findings provide support for our GABA hypothesis, it is important to consider whether the corticomotor inhibition observed in the current study could have been increased by factors other than subconcussive impacts. For example, cSP increases have been measured during a fatiguing exercise or contraction (Taylor et al., 1996; Søgaard et al., 2006). Increased inhibitory mechanisms following sparring, coupled with decreased MVC, could therefore be interpreted as evidence that corticomotor inhibition relates to muscular fatigue. We believe this to not be the case due to a number of reasons. First, MCV values in both sparring and control group decreased in a similar fashion, whilst cSP only increased following sparring. Second, whilst cSP increases following muscle-specific fatiguing exercises, it appears to either be unchanged/suppressed following moderate/high intensity whole body exercises (O’Leary et al., 2016) such as the activity in the current study. Third, cSP increases associated with fatigue are short-lived, returning to pre-exercise vales almost immediately upon completion of the exercise (Taylor et al., 1996; Søgaard et al., 2006), while the effects in this study were delayed. As such, we suggest that the increases in cSP in the current study most likely reflect heightened GABAergic activity resulting from repetitive subconcussive head impacts. In light of these findings, future research should endeavor to quantify actual changes to GABA concentrations following routine impact in sport through magnetic resonance spectroscopy.

**FIGURE 5:**
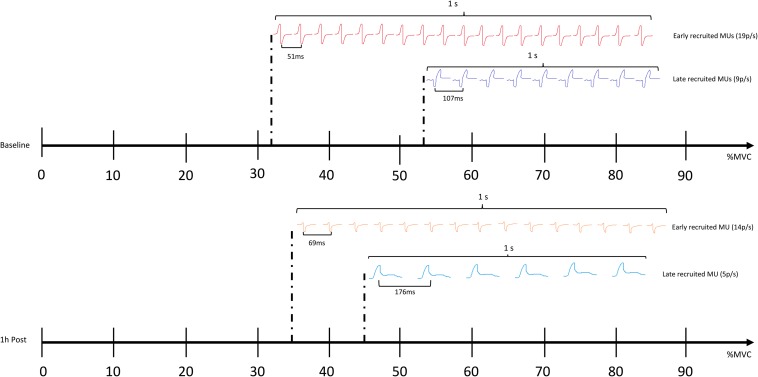
Comparing the recruitment threshold (%MVC) of the individual motor units (MUs) against their average firing rate (pulses per second – p/s) using a linear regression shows a negative shift in the regression line 1 h following sparring. The finding suggests that the recruitment patterns and the firing rates of the MUs as a whole are altered; the early recruited, small motor units are activated later compared to baseline, and the frequency with which they propagate on the sarcolemma of the muscle is slowed down (showed by an increase in the average inter-pulse interval – IPI, and a decrease in how many pulses are discharged per second). The later recruited, big motor units are activated earlier compared to baseline, yet also show an increase in IPI and a decrease in the pulses discharged per second.

GABA mediated inhibition is critical for motor function, as recently demonstrated by modulation of inhibitory neurotransmitter receptors in leg motoneurons, leading to changes in gait (Gowda et al., 2018). Cortical inhibition is thought to modulate muscle function by dampening corticospinal input (Motawar et al., 2012). As predicted, increased GABA activity caused by the impacts incurred from sparring in the current study, appears to have diminished the descending neural drive to the muscles, affecting motor unit action potentials propagation from central to peripheral systems. This mechanism is reflected by altered recruitment thresholds of motor units occurring, alongside increased TMS assessed corticomotor inhibition (1 h post), with low threshold motor units being activated later and high threshold motor units being engaged earlier compared to baseline (Figure 5). As such, these changes to motor unit behavior, coupled with increased corticomotor inhibition, further support the idea that a GABAergic response (also seen in concussed patients) may be triggered following subconcussive blows to the head. Other changes to motor control, such as postural control, are commonly found to be affected following concussion (e.g., as part of standard concussion assessment). Effects of repetitive subconcussive head impacts on postural control has shown diverging results, with studies showing decrements (Haran et al., 2013; Hwang et al., 2017), and others showing no change (Broglio et al., 2004; Di Virgilio et al., 2016). Postural control is a fairly indirect measure of motor control and may not be sensitive enough to pick up subtle changes to brain function resulting from repetitive subconcussive head impacts; it is not surprising, therefore, that postural control appeared unchanged in the current study.

The assessment of motor unit behavior changes following routine subconcussive impact in sport also has important practical implications. Earlier recruitment of high threshold motor units may also explain potential compensatory effects for sustaining fatigue-reduced maximum voluntary contractions similar to this study’s control group. This is an interesting finding given the suggestion that reduced motor unit recruitment and firing patterns likely reflect diminished descending neural drive from the CNS during times of stress or danger (Nybo and Nielsen, 2001; Hunter et al., 2011).

Disrupted inhibitory and excitatory cortical pathways are a possible reason for increased musculoskeletal injury rates following concussion, reflecting an impairment in the ability to effectively exert control over the muscles (Lynall et al., 2015; Cross et al., 2017). The pattern of results in the current study suggests that repetitive subconcussive head impacts could increase the risk of musculoskeletal injury through similar mechanisms to those observed following concussive injuries. To the best of our knowledge, this is the first study to integrate the use of TMS and dEMG in the context of head impacts, showing possible changes at the cerebral level translating to changes at a muscular level.

Dampened corticospinal excitability 24 h post-sparring was unexpected, as based on our previous findings (Di Virgilio et al., 2016) and published literature (Miller et al., 2014; Pearce et al., 2015) we predicted that this excitatory parameter would remain unchanged. One potential explanation of this finding is that excitation increased slightly (though not significantly) in response to the increased inhibition 1 h post-sparring, and the decreased excitation at the 24 h follow-up is a compensatory mechanism to this acute response. However, due to the lack of data on the relationship between excitation and subconcussive impacts, and the divergent results from concussion literature (Chistyakov et al., 2001; Livingston et al., 2010; Miller et al., 2014; Pearce et al., 2015) more research on excitation-concussion/subconcussion is needed before any informed conclusions can be made.

Finally, we highlight that an important target for future research is to examine the relationship between the amount of impact and inhibitory change. Because we used real sparring sessions, in the current study it was not possible to control the force of impact experienced by each boxer. From this perspective a limitation of the current findings is that we did not quantify the impact exposure or the dose-response link between impact incurred and the effects found. A further limitation of the current study is that we did not distinguish between impacts to the body or to the head. As such, we cannot discern to what extent the observed effects arise from direct impacts to the head (as in a soccer heading paradigm) or indirectly, where head movement occurs as a consequence of impacts to the body.

Following the findings of this study demonstrating that routine impact in sport gives rise to brain changes, a key aim for future studies is to assess the dose-response curve, characterizing the degree of inhibitory change as a function of the force of impact, for example by implementing the use of telemetry data and TMS in a subconcussive context. Most importantly, perhaps, having established that increased brain health and behavior are affected following subconcussive head impacts in both football and boxing, it is critically important to understand the limits of impact exposure producing a GABAergic response in order to determine how much impact is safe.

## Conclusion

Using a multi-methods approach this study characterizes acute brain alterations occurring following routine head impacts in sport suggestive of a GABAergic response. One hour after sparring boxers showed increased corticomotor inhibition, altered motor unit recruitment strategies, and decreased memory performance relative to controls. Although transient, these brain changes are reminiscent of effects seen following brain injury, and suggest a potential mechanism underlying the damaging long-term effects of routine repetitive head impacts in sport. Dampening of motor control following routine impact, as first demonstrated here, has practical implications that athletes may be at greater risk of musculoskeletal injuries following routine sport-related (head) impacts.

## Data Availability

The datasets generated for this study are available on request to the corresponding author.

## Ethics Statement

All subjects gave written informed consent in accordance with the Declaration of Helsinki. The protocol was approved by the School of Sport Research Ethics Committee (SSREC number 860) at the University of Stirling.

## Author Contributions

TD, MI, LW, DD, and AH conceived and designed the study, interpreted the data, and made substantial contributions to the revisions of the manuscript prior to the submission. TD performed the data collection and analysis. TD, MI, and AH prepared the first draft of the manuscript.

## Conflict of Interest Statement

The authors declare that the research was conducted in the absence of any commercial or financial relationships that could be construed as a potential conflict of interest.
